# Rapidly Recurrent Exotropia After Surgery Unmasks an Oculomotor Nerve Meningioma: A Diagnostic Pitfall

**DOI:** 10.1002/kjm2.70300

**Published:** 2026-10-12

**Authors:** Ting‐En Chen, Hsiu‐Mei Huang, You‐Ting Wu, Jung Lo

**Affiliations:** ^1^ School of Medicine, College of Medicine Kaohsiung Medical University Kaohsiung Taiwan; ^2^ Department of Ophthalmology Kaohsiung Chang Gung Memorial Hospital and Chang Gung University College of Medicine Kaohsiung Taiwan; ^3^ Department of Laboratory Medicine and Department of Anatomic Pathology Chiayi Chang Gung Memorial Hospital and Chang Gung University College of Medicine Chiayi Taiwan; ^4^ Graduate Institute of Clinical Medicine, College of Medicine Kaohsiung Medical University Kaohsiung Taiwan

Recurrent exotropia (XT) is a recognized outcome after strabismus surgery, but its frequency and timing vary considerably across exotropia subtypes [1]. Early or rapidly progressive recurrence accompanied by neurological signs should raise suspicion for an underlying neurological disorder. Intracranial masses involving cranial nerves are uncommon but critical causes of secondary strabismus. We report a diagnostic pitfall in which rapidly recurrent XT masked an undiagnosed oculomotor nerve (cranial nerve III) meningioma.

A 25‐year‐old man presented to an outside hospital with a 3‐year history of progressive right XT and binocular diplopia. Examination revealed a constant right XT of 30 prism diopters (PD) with mildly limited adduction (−1); neither ptosis nor pupillary abnormalities were documented. He subsequently underwent right lateral rectus recession (7.0 mm) and medial rectus plication (5.5 mm), designated as Month 0. At Month 1, XT improved to 14 PD, although the deviation rapidly recurred and worsened by Month 4. At Month 16, he presented to our hospital with 65 PD of right XT. Visual acuity was 20/20 in both eyes. Mild right‐sided ptosis, a poorly reactive pupil, and restricted ocular motility in adduction (−4), elevation (−3), and depression (−3) with completely preserved abduction (0) were noted (Figure 1A–I). The exotropia was incomitant, with maximal deviation in left gaze, suggesting right oculomotor nerve palsy. Serologic tests showed normal thyroid function and were negative for anti‐acetylcholine receptor antibodies. Contrast‐enhanced brain magnetic resonance imaging performed at Month 17 demonstrated a 7‐mm avidly enhancing extra‐axial lesion along the lateral margin of the right oculomotor cistern, abutting the right rostral cavernous sinus, findings highly suggestive of an oculomotor nerve tumor (Figure 1J,K). Magnetic resonance angiography showed no evidence of aneurysm or other vascular abnormalities. At Month 19, a right pterional craniotomy demonstrated a tumor concentrically involving the oculomotor nerve, with atrophy of the nerve distal to the lesion. Because nerve‐sparing resection was not feasible and partial resection carried a risk of tumor recurrence or progression, the tumor and involved nerve segment were resected en bloc, leaving an approximately 15‐mm nerve defect that was reconstructed with a sural nerve graft. Histopathological examination showed fascicles of meningothelial cells with oval nuclei and eosinophilic cytoplasm. Immunohistochemical staining was positive for epithelial membrane antigen (Figure 1L), focal S‐100, vimentin, a Ki‐67 index of 2%, and negative for glial fibrillary acidic protein, confirming a World Health Organization Grade 1 meningioma. At the final follow‐up at Month 31, there was no tumor recurrence; however, complete right ptosis and approximately 60 PD of XT persisted. Further neurological recovery remains uncertain, although partial functional recovery after sural nerve reconstruction has been reported in oculomotor nerve meningioma [2]. Additional strabismus surgery and frontalis suspension were considered but deferred because the current ptosis reduces symptomatic diplopia, whereas eyelid correction may result in lagophthalmos and unmask diplopia.

**FIGURE 1:**
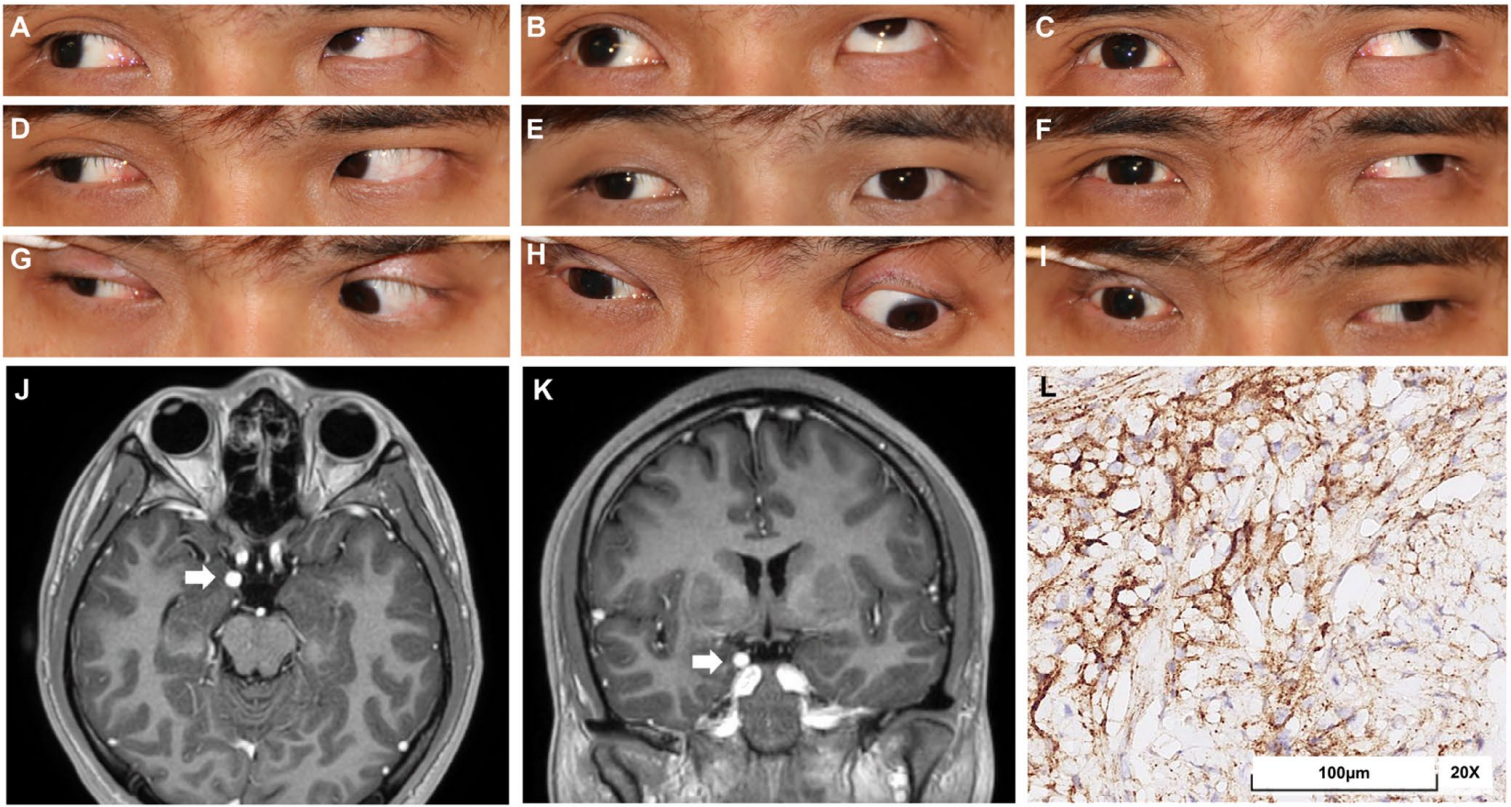
Multimodal diagnostic findings of oculomotor nerve meningioma. (A–I) Nine diagnostic positions of gaze obtained 1 year after primary strabismus surgery of the right eye. Prism cover testing demonstrated 65 prism diopters (PD) of right exotropia in the primary position. The photographs demonstrate limitation of right ocular movements in all directions except abduction, accompanied by mild right blepharoptosis. (J, K) Contrast‐enhanced axial and coronal T1‐weighted magnetic resonance images demonstrate a 7‐mm avidly enhancing lesion (white arrow) along the lateral margin of the right oculomotor cistern, abutting the right cavernous sinus. (L) Immunohistochemical staining of the resected tumor displays strong positivity for epithelial membrane antigen (EMA) (magnification 20 ×), confirming the diagnosis of a World Health Organization (WHO) grade 1 meningothelial meningioma. Scale bar = 100 μm.

Meningiomas arising from cranial nerves without dural attachment are uncommon and may remain undiagnosed until neurological deficits manifest [3, 4]. This patient illustrates how an intracranial tumor can initially masquerade as isolated strabismus, particularly when early neuro‐ophthalmic signs are subtle. Importantly, a deviation that rapidly recurs and eventually exceeds the preoperative measurement is atypical after standard strabismus surgery and should raise suspicion for a secondary etiology. Recurrent exotropia after strabismus surgery is usually related to mechanical or sensory factors, including insufficient surgical dosage, amblyopia, or poor binocular fusion [1]. Conversely, the rapid progression in our patient, with the deviation increasing from 30 to 65 PD and accompanied by extraocular motility limitation, ptosis, and pupillary abnormalities, was highly atypical and suggested an underlying neurological lesion, warranting prompt neuroimaging [5]. The differential diagnosis of a small enhancing lesion along oculomotor nerve includes schwannoma and meningioma. In the absence of a characteristic dural attachment, their imaging features may overlap [4], as in our patient, in whom the diagnosis of meningioma was ultimately confirmed histopathologically. Early diagnosis is important because selected small meningiomas may be managed with stereotactic radiosurgery, potentially preserving nerve function [3]. Once neurological deficits progress, however, surgical resection may become necessary to obtain a histological diagnosis, relieve mass effect, and achieve durable local tumor control [3]. When the oculomotor nerve is circumferentially involved by the tumor, nerve preservation may not be feasible. Functional recovery after sural nerve reconstruction remains uncertain; partial recovery has been reported, although persistent deficits may occur [2].

In conclusion, this case highlights the importance of considering neuroimaging before strabismus surgery in adults with acquired progressive strabismus, incomitancy or unexplained motility restriction, or associated neuro‐ophthalmic signs, as well as in patients with rapid or atypical strabismus recurrence. Early recognition may help avoid unnecessary strabismus surgery, reduce diagnostic delay, and facilitate timely multidisciplinary management.

## Funding

This study was supported by the grants from National Science and Technology Council, Taiwan (Grant number: NSTC 113‐2221‐E‐182A‐001‐MY3).

## Ethics Statement

This study was approved by the Institutional Review Board of Chang Gung Medical Foundation (202601557B0).

## Conflicts of Interest

The authors declare no conflicts of interest.

## Supporting information


**Figure S1:** Clinical timeline of the patient's disease course. Following transient improvement after initial strabismus surgery (Month 0), the right exotropia rapidly recurred and progressed, leading to neuroimaging and subsequent diagnosis of an oculomotor nerve meningioma. The tumor was excised at Month 19 with oculomotor nerve reconstruction using a sural nerve graft, and no tumor recurrence was detected at the 1‐year postoperative follow‐up (Month 31). LR, lateral rectus; MR, medial rectus; MRI, magnetic resonance imaging; PD, prism diopters.

## Data Availability

The data that support the findings of this study are available from the corresponding author upon reasonable request. Written informed consent for publication was obtained from the patient.
